# Renal microthrombosis and thrombomodulin deficiency in COVID-19–associated acute kidney injury

**DOI:** 10.1186/s12882-026-04788-2

**Published:** 2026-01-31

**Authors:** Matilda Bekhet, Amanda Marks-Hultström, Gül Gizem Korkut, Angelina Schwarz, Jaakko Patrakka, Elisabet Englund, Marie Jeansson

**Affiliations:** 1 Department of Medicine Huddinge, Karolinska Institutet, Blickagangen 16, Huddinge, Sweden; 2 Department of Immunology, Genetics, and Pathology, Uppsala University, Uppsala, Sweden; 3 Department of Laboratory Medicine, Karolinska Institutet, Huddinge, Sweden; 4 Department of Clinical Sciences, Karolinska Institutet, Huddinge, Sweden; 5 Division of Pathology, Department of Clinical Sciences, Lund University, Lund, Sweden

**Keywords:** Acute kidney injury, Angiopoietin-2, COVID-19, Thrombomodulin, Thrombosis

## Abstract

**Background:**

Severe COVID-19 frequently involves multi-organ dysfunction, including acute kidney injury (AKI), which affects up to 85% of critically ill patients. While both direct viral infection and systemic effects are implicated, the role of renal microthrombosis remains poorly defined in COVID-19 and AKI. Angiopoietin-2, a pro-inflammatory cytokine, and cleaved thrombomodulin is elevated in plasma in severe COVID-19 and has been linked to endothelial dysfunction and hypercoagulability. We hypothesize that renal microthrombi can contribute to decreased kidney function in critically ill COVID-19 patients.

**Methods:**

We performed histopathological and molecular analyses of postmortem kidney tissue from seven critically ill COVID-19 patients. Control tissue was obtained from nephrectomy specimens (*n* = 6) and postmortem tissue (*n* = 7). We assessed microthrombi, tubular necrosis, glomerulosclerosis, fibrosis, and expression of angiopoietin-2 and thrombomodulin. Immunofluorescence and SARS-CoV-2 nucleoprotein staining were used alongside clinical data.

**Results:**

AKI was observed in six of seven COVID-19 patients. Compared to controls, COVID-19 kidneys showed a significant reduction in tubular nuclear area (*P* < 0.0003), presence of viral antigen in tubular epithelium, and marked glomerular and peritubular microthrombi (15.2 vs. 1.3 thrombi/mm²; *P* < 0.0001). THBD expression was significantly reduced bith peritubular capillaries and glomeruli in COVID-19 kidneys. Glomerulosclerosis, glomerular area, and tubulointerstitial fibrosis were variable in both control and COVID-19 patients with no significant differences.

**Conclusions:**

This study identifies widespread renal microthrombi, tubular necrosis, and reduced THBD expression in COVID-19 patients with AKI, supporting a role for endothelial dysfunction and microvascular thrombosis in COVID-19-associated renal injury. The data implicates the disruption of endothelial anticoagulant signaling through thrombomodulin as a contributing mechanism.

## Introduction

Severe acute respiratory syndrome coronavirus 2 (SARS-CoV-2), the causative agent of Coronavirus Disease 2019 (COVID-19), exhibits a wide clinical spectrum ranging from asymptomatic infection to severe disease requiring intensive care unit admission. Although the respiratory system is the primary site of viral infection, COVID-19 is now recognized as a systemic, multi-organ disease characterized by widespread inflammation, endothelial dysfunction, coagulopathy, hypoperfusion, and multi-organ failure.

Acute kidney injury (AKI) occurs in up to 85% of critically ill patients with COVID-19 [1–4]. AKI is a heterogeneous syndrome ranging from transient functional impairment to complete renal failure requiring renal replacement therapy [5]. Both direct and indirect mechanisms have been implicated in COVID-19-associated AKI. Direct renal injury may result from viral entry into renal cells, complement activation, and localized inflammation [6]. Indirect mechanisms are likely to be more prevalent and include systemic inflammation, sepsis, thromboembolic disease secondary to hypercoagulability, and exposure to nephrotoxic agents [6]. Several studies have identified thrombi in glomerular and peritubular capillaries of COVID-19 kidneys [7–10], whereas others have failed to detect thrombotic lesions [11]. Importantly, AKI is associated with increased risk of long-term complications, including progression to chronic kidney disease (CKD), even among COVID-19 survivors [12]. A deeper understanding of the pathophysiological mechanisms underlying AKI in severe COVID-19 is therefore essential for improving clinical management and outcomes.

We have previously demonstrated a mechanistic role for the proinflammatory cytokine angiopoietin-2 (ANGPT2) in promoting hypercoagulability in vivo [13]. ANGPT2 inhibits thrombomodulin (THBD)-mediated activation of protein C, thereby disrupting a key endogenous anticoagulation pathway [13, 14]. THBD is constitutively expressed on the luminal surface of vascular endothelial cells and plays a critical role in the anticoagulant and anti-inflammatory properties of the endothelium [15, 16]. Notably, exogenous administration of ANGPT2 in animal models leads to shedding of endothelial THBD, resulting in elevated circulating levels of the protein [13]. In patients with severe COVID-19, plasma levels of both ANGPT2 and THBD are elevated and correlate with disease severity and mortality [13, 17–21]. We recently reported a strong association between elevated ANGPT2 levels, hypercoagulability, and impaired renal function in critically ill COVID-19 patients [13]. In addition to its anticoagulant-disrupting effects, ANGPT2 is a well-established antagonist of the endothelial-specific TIE2 receptor [22]. Under normal physiological conditions, TIE2 activation by its ligand angiopoietin-1 (ANGPT1) promotes endothelial quiescence and vascular integrity [23]. ANGPT2 antagonism of TIE2 signaling leads to endothelial destabilization, vascular leakage, inflammation, and further induction of ANGPT2 transcription, establishing a pathogenic feed-forward loop [24–30]. These mechanisms likely contribute to the widespread endothelial dysfunction observed in COVID-19.

Based on these findings, we hypothesize that AKI in critically ill COVID-19 patients may be caused in part by renal microthrombi. To investigate this, we performed a detailed histopathological and molecular analysis of postmortem kidney tissue from seven critically ill COVID-19 patients, a patient group previously identified as having high circulating ANGPT2 levels [13]. Control kidney tissue was obtained from histologically normal areas of nephrectomy specimens from patients undergoing surgery for kidney cancer (*n* = 6) and postmortem specimens (*n* = 7) from patients without COVID-19. We assessed the presence of renal microthrombi, tubular necrosis, tubulointerstitial fibrosis, glomerulosclerosis, and protein expression of ANGPT2 and THBD in both groups, aiming to clarify the contribution of microvascular to COVID-19-associated AKI.

## Methods

### Study design and patients

This study analysed postmortem renal tissue and corresponding clinical data from critically ill patients with COVID-19. The study was approved by the Swedish Ethical Review Authority (EPM; approval numbers 2021–03959 and 2022-03936-02). Informed consent was obtained from the patients themselves or, when not possible, from their next of kin. All procedures adhered to the principles of the Declaration of Helsinki and its subsequent amendments. Clinical data were reported in accordance with the STROBE (Strengthening the Reporting of Observational Studies in Epidemiology) guidelines.

All included patients were deceased individuals with COVID-19 who received intensive care during hospitalization in 2020 or 2021. Inclusion criteria were a confirmed diagnosis of COVID-19 by reverse-transcription polymerase chain reaction (RT-PCR) from nasopharyngeal swabs and treatment in the intensive care unit (ICU). AKI was not a criterion for inclusion. In total, renal tissue samples from seven patients were analysed. Patients were anonymized and assigned numerical identifiers (C19-1 to C19-7) unrelated to identifiable personal information outside the research team.

Postmortem kidney samples were obtained from the Region Syd Biobank. The interval between death and autopsy ranged from 4 to 7 days, during which the bodies were kept in cold storage. For two patients (C19-2 and C19-5), duplicate samples were obtained to assess intra-individual variability. These replicates yielded consistent results and were included in patient-level averages. Control kidney tissue was obtained from histologically normal appearing areas of nephrectomy specimens from patients undergoing surgery for renal malignancy (*n* = 6) and from postmortem tissue (*n* = 7) from patients without COVID-19. All samples were formalin-fixed and paraffin-embedded (FFPE).

Clinical data for the COVID-19 cohort were extracted from the digital health record system Melior and included demographics, medical history, and biochemical markers (C-reactive protein [CRP], interleukin-6 [IL-6], ferritin, D-dimer, fibrinogen, platelet count, plasma creatinine, estimated glomerular filtration rate [eGFR], and troponin T). Clinical data were not available for the control group.

## Kidney histology and microthrombosis

Histological evaluation was performed on FFPE kidney sections stained with Masson’s Trichrome and fibrinogen (A0080, Dako), processed at Uppsala University Hospital, Department of Clinical Pathology. Due to extensive background staining likely caused by necrotic tubular epithelial cells, fibrinogen staining was excluded from quantitative analysis.

Additional histological assessment included Martius Scarlet Blue (MSB) staining (Atomic Scientific, RRSK2-200) following the manufacturer’s protocol. Slides were scanned using a Zeiss AxioScan slide scanner at 20× magnification, and images were analysed using QuPath v0.4.0.

Thrombi and sclerotic glomeruli were manually quantified and expressed as thrombi/mm² and sclerotic glomeruli/total glomeruli, respectively. More than 4,000 glomeruli were assessed in total. The analysed tissue area ranged from 33 to 214 mm². Glomerular area and tubulointerstitial fibrosis were measured in all samples. Fibrosis was quantified on Masson’s Trichrome-stained sections as the fibrotic area relative to the total tissue area.

### Immunohistochemistry for nuclei, angiopoietin-2, thrombomodulin, pecam1, and SARS-CoV-2

Immunofluorescence staining was performed on 10-µm paraffin sections. Tissue sections underwent deparaffinization with xylene and a graded alcohol series (100%, 70%, 50%) followed by heat-induced antigen retrieval (2369, DAKO) at 95 °C for > 20 min in pH 9 buffer. Samples were blocked with serum-free blocking buffer (X0909, DAKO) supplemented with 0.25% Triton X-100 (T9284, Merck) for one hour at room temperature.

Primary antibodies were applied at 1:100 dilution and incubated overnight at 4 °C. The antibodies used included goat anti-angiopoietin-2 (R&D Systems, AF623), rabbit anti-thrombomodulin (Invitrogen, PA5-120883), and mouse anti-PECAM1 (Abcam, Ab949850D). After washing, samples were incubated with Alexa Fluor-conjugated secondary antibodies (Thermo Fisher Scientific; 1:100 dilution) for two hours at room temperature: donkey anti-rabbit IgG (A10043 or A31572), donkey anti-goat IgG (A21432), and donkey anti-mouse IgG (A10038 or A21202). Nuclear counterstaining was performed with Hoechst 33,342 (Life Technologies, H3570), and slides were mounted using ProLong Gold Antifade Mounting (P36930, Thermo Fisher Scientific). Images were acquired using a Leica SP8 confocal microscope at 400× magnification. Both renal cortex and PECAM1-positive glomeruli (to exclude sclerotic glomeruli) were imaged. Quantification of nuclear, ANGPT2, and THBD staining was performed using ImageJ (NIH). The stained area was expressed as a percentage of the total image area. For each sample, 5–15 images of both glomeruli and renal cortex were averaged for group-level comparisons. Loss of nuclear staining was used as an indicator of tubular cell integrity, with loss suggestive of necrosis.

For SARS-CoV-2 staining, rehydration and antigen retrieval was performed as above on paraffin sections. Endogenous peroxidase was quenched with 3% hydrogen peroxide in methanol for 30 min. To eliminate background staining from endogenous biotin, sections were treated with an avidin/biotin blocking kit (SP-2100, Vector Laboratories) according to the manufacturer’s instructions. Sections were then blocked with 10% goat serum for 30 min at room temperature. Slides were incubated with SARS-CoV-2 Nucleocapsid Monoclonal Antibody (B46F) (MA1-7404, Thermo Fisher Scientific) at a dilution of 1:20 for 1 h at room temperature. Slides were washed and subsequently incubated with a biotinylated rabbit anti-mouse IgG antibody (E0354, Dako) for 1 h at room temperature. The signal was amplified using Vectastain ABC reagent (PK-6100, Vector Laboratories) until optimal color development (3 min). Sections were mounted with Vectamount Express mounting medium (H-5700, Vector Laboratories). Images were acquired using a Zeiss Axiolab upright brightfield microscope equipped with a Zeiss Axiocam 208c camera at 200× magnification.

### Proximity ligation assay

To investigate protein-protein interactions between ANGPT2 and THBD, a proximity ligation assay (PLA) was performed, targeting molecular interactions within < 40 nm distance. The same primary antibodies used for immunofluorescence were employed. The PLA was conducted using the Duolink^®^ system (DUO02021) with in situ detection reagents (DUO92008, Sigma-Aldrich), following the manufacturer’s protocol. The secondary antibodies included donkey anti-rabbit IgG PLUS (DUO092002, Merck) and donkey anti-goat IgG MINUS (DUO92006, Merck). Mounting and imaging procedures were performed as previously described.

### Statistics

All data are presented as median with interquartile range (IQR). Group comparisons were conducted using Mann-Whitney test in GraphPad Prism version 10 (GraphPad Software Inc.). Spearman correlation coefficients were used to evaluate correlation. A *P*-value < 0.05 was considered statistically significant.

## Results

### Patient characteristics

Demographic and clinical characteristics of the study participants are summarized in Table 1. The cohort consisted of seven critically ill patients with confirmed COVID-19, including two females and five males. All patients succumbed to the disease between 4 and 26 days post-hospitalization, with a mean duration of 11 days. The median age at the time of death for COVID-19 patients was 72 (63–75) years. The median age of patients providing postmortem control tissue was 73 (57–81) years, including two females and five males. No information was available for control tissue. 

**Table 1:** Patient demographics and clinical parameters

Gender (F/M)	Reference range	All patients	C19-1	C19-2	C19-3	C19-4	C19-5	C19-6	C19-7
			F	F	M	M	M	M	M
Age (years)		72 (63–75)							
BMI (kg/m^2^)		27.8 (23.5–31.4)							
Hospital time (days)		12 (6–20)							
Invasive ventilation (Y/N)			Y	Y	ECMO	N	Y	N	Y
Hypotension/vasopressor			Y	Y	Y	N	Y	N	Y
Steroids			Y	Y	Y	Y	Y	N	Y
Antivirals			Y	Y	N	N	N	N	Y
Renal replacement therapy			N	N	CRRT*	N	N	N	N
Thromboprophylaxis			Y	Y	Y	Y	Y	Y	Y
History of HT, DM, CKD, tumor, RA, TE, COPD			HT COPD	RA	N	N	HT Tumor TE	HT DM CKD5	HT DM TE COPD
Cause of death			RF	CH	RF, H	sepsis	MI	MOF	MOF
PaO_2_/FiO_2_	> 60	8 (7.5–18.5)	8	8	11	*n/a*	7	*n/a*	26
CRP (mg/l)	< 5 mg/l	176 (76–335)	97	188	29	176	473	76	335
IL6 (ng/l)	< 7.0 ng	380 (123–5988)	78	138	8639	258	503	*unk*	5104
Ferritin (µg/l)	F: 13–330; M: 30–400	1122 (808–1513)	808	1122	1513	552	1122	1236	2248
D-dimer (mg/l FEU)	< 0.5	2.4 (1.7–24.0)	3.7	2.4	> 40	24.0	1.7	1.6	2.2
Fibrinogen	2.0-4.2	9.0 (4.2-9.0)	4.3	> 9.0	> 9.0	4.2	> 9.0	4.1	> 9.0
Platelets (x10^9^/l)	150–350	149 (62–225)	225	282	208	71	149	61	62
Hb (g/l)	F: 120–150; M:130–170	95 (90–137)	149	95	90	137	92	82	110
Creatinine (µmol/l)	F:45–90, M: 60–105	159 (134–320)	170	138	159	69	320	517	134
eGFR (ml/min/1.73 m^2^)	Age > 65: 50–90	27 (20–49)	30	23	33	93	20	10	25
Troponin T (ng/l)	< 15	86 (41–149)	138	*unk*	*unk*	40	42	86	160

Data are expressed as median (IQR). BMI, body mass index; ECMO, extracorporeal membrane oxygenation; CRRT*, continuous renal replacement therapy due to sepsis; N, none; HT, hypertension; DM, diabetes mellitus type 2; CKD, chronic kidney disease (stage); RA, rheumatoid arthritis; TE, thromboembolism; COPD, chronic obstructive pulmonary disease; RF, respiratory failure; CH, cerebral hemorrhage; H, hemorrhage; MI, myocardial infarction; MOF, multi organ failure; PaO_2_/FiO_2_, the ratio of arterial oxygen partial pressure (PaO_2_ in mmHg) to fractional inspired oxygen; Hb, hemoglobin; eGFR, estimated glomerular filtration rate from P-creatinine; *unk*, unknown; *n/a*, not applicable

Five of the seven COVID-19 patients had preexisting comorbidities of varying severity, including hypertension, diabetes mellitus, chronic kidney disease, pulmonary disorders, and a history of thromboembolic events. None of the patients had a history of severe heart failure. Two individuals had no known comorbidities at the time of COVID-19 diagnosis. One patient developed sepsis prior to death. All patients received treatment in an intensive care unit (ICU). Four patients underwent invasive mechanical ventilation, and one received extracorporeal membrane oxygenation (ECMO) (Table 1). Clinical data were not available for individuals providing control kidney tissue samples were obtained.

### AKI occurred in a majority of critically ill COVID-19 patients

In this study, 6 out of 7 patients with severe COVID-19 developed AKI during their hospitalization, evidenced by elevated plasma creatinine (P-creatinine) levels and decreased estimated glomerular filtration rate (eGFR) (Table 1). One patient (C19-4) exhibited normal renal function and had no history of comorbidities prior to SARS-CoV-2 infection. Another patient (C19-6) had preexisting chronic kidney disease stage 5 (CKD5), with an eGFR of 10 ml/min/1.73 m². The remaining patients had no documented history of renal impairment before their COVID-19 diagnosis.

**Fig. 1:**
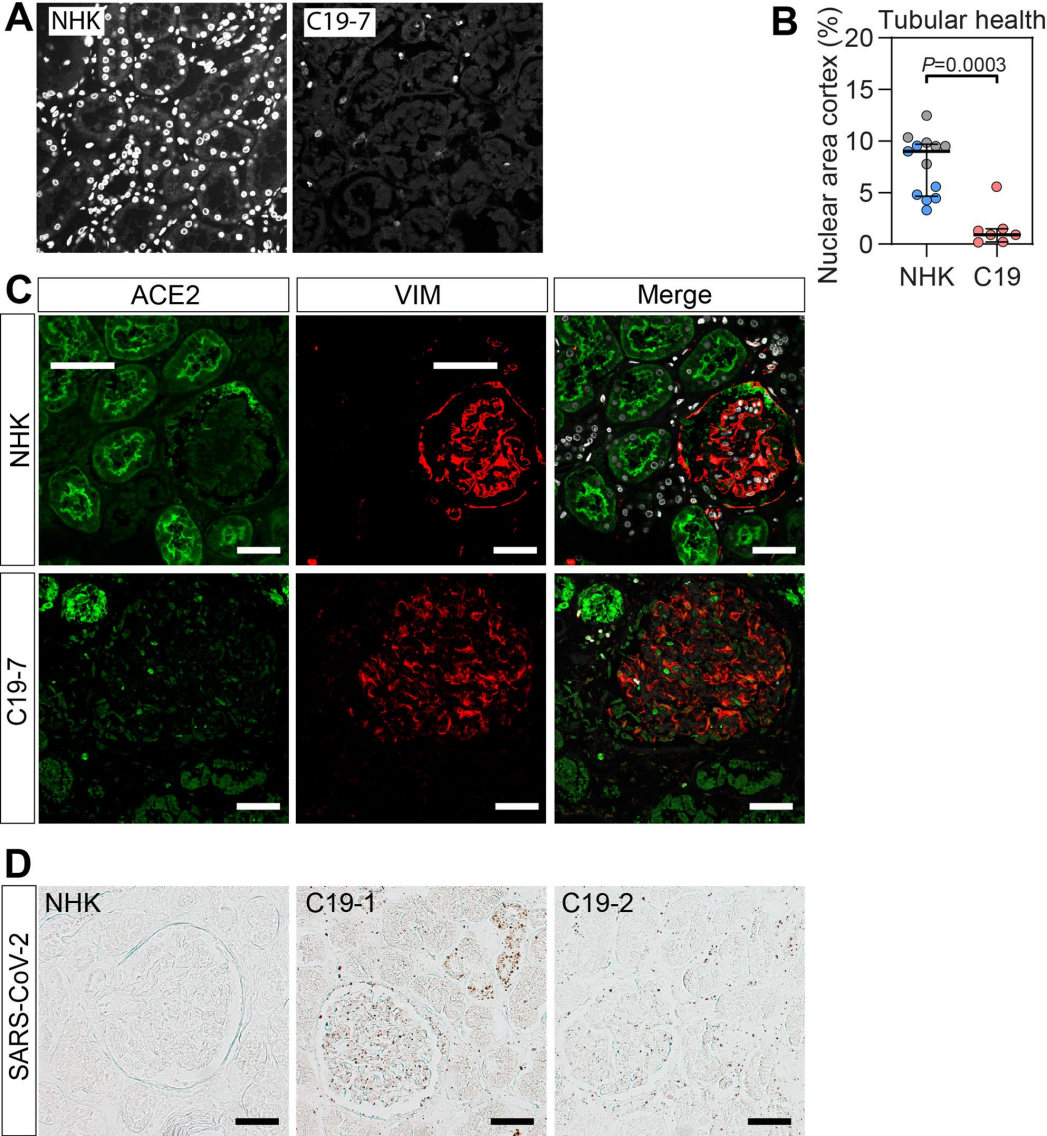
Abundant tubular necrosis in critically ill COVID-19 patients. (**A**) Nuclear staining with Hoechst of renal cortex samples from normal human kidneys (NHK) obtained from nephrectomy specimens (*n* = 6, grey), postmortem controls (*n* = 7, blue), and kidneys from COVID-19 patients (C19, *n* = 7, red). (**B**) The area of nuclear staining was quantified and used as a measure of tubular cell integrity. (**C**) Fluorescence immunohistochemistry of ACE2 (green) and vimentin (VIM, red) expression in renal cortex from postmortem normal human kidney (NHK) and COVID-19. Vimentin is a marker of podocytes and mesenchymal cells. Original magnification x400, scale bars = 50 μm. (**D**) SARS-CoV-2 staining (brown dots) in renal cortex from postmortem normal human kidney (NHK) and two COVID-19 patients. Data are presented as median (IQR). Statistical analysis was performed using the Mann Whitney test

To assess tubular epithelial integrity, nuclear area was quantified using Hoechst staining of confocal images from renal cortical tissue, excluding glomeruli. Control kidney tissue demonstrated a mean nuclear area of 9.0%, which was significantly reduced to 0.9% in kidneys from COVID-19 patients (*P* < 0.0003; Fig. 1A, B; Table 2). A proposed mechanism for the observed tubular necrosis is direct viral entry into tubular epithelial cells via the angiotensin-converting enzyme 2 (ACE2) receptor [31]. Immunohistochemical staining for ACE2 revealed tubular luminal localization in control kidney tissue whereas COVID-19 patients had fewer positive tubular segments but staining throughout the cell (Fig. 1C). SARS-CoV-2 staining was confirmed in renal tissue of COVID-19 patients, including the glomerulus, some tubular segments, and the interstitium (Fig. 1D). The extensive tubular necrosis in COVID-19 patients makes interpretation of ACE2 and SARS-CoV-2 staining difficult.

**Table 2:** Postmortem kidney data from COVID-19 patients and controls data are expressed as median (IQR). *P*-value is comparison of C19 with the combined NHK group using Mann-Whitney test. ANGPT2, angiopoietin-2; THBD, thrombomodulin

Thrombi/mm^2^	NHK nephrectomy (*n* = 6)	NHK post-mortem (*n* = 4)	C19 patients (*n* = 7)	*P*-value (C19 vs. all NHK)	C19-1	C19-2	C19-3	C19-4	C19-5	C19-6	C19-7
	0.0 (0-2.1)	1.5 (0.3–2.4)	15.2 (7.5–19.6)	0.0001	15.2	27.8	17.9	1.9	7.6	12.9	19.6
Tubular health (% nuclear area)	9.7 (9.1–10.9)	4.8 (4.3-9.0)	0.9 (0.2–1.5)	0.0003	0.9	0.9	0.2	1.3	1.5	5.6	0.2
Glomerulosclerosis (%)	0 (0-3.8)	11.2 (6.8–17.0)	9.9 (3.7–19.8)	ns	6.5	19.8	0.9	9.9	34.9	18.7	7.3
Glomerular size (x10^3^ µm^2^)	18.7 (15.3–25.6)	24.9 (17.6–25.3)	25.3 (22.7–30.6)	ns	23	18	35	31	24	25	28
Tubulointerstitial fibrosis (%)	31.0 (18.1–46.9)	40.4 (31.8–45.2)	34.5 (27.7–56.0)	ns	27.7	27.9	37.6	22.6	66.5	61.6	19.7
ANGPT2 glomeruli (%)	0.8 (0.5–1.5)	0.4 (0.2–0.5)	0.7 (0.6–1.4)	ns	1.4	0.6	0.8	0.2	0.7	1.2	0.6
ANGPT2 cortex (%)	0.1 (0.1–0.3)	0.2 (0.1–0.4)	0.2 (0.1–0.7)	ns	0.3	0.1	1.2	0.1	0.2	0.7	0.2
THBD glomeruli (%)	2.8 (1.7–3.2)	2.4 (1.9–3.2)	0.8 (0.6–1.4)	0.0017	0.6	0.8	0.1	1.4	1.7	2.2	1.2
THBD cortex (%)	1.7 (0.9–3.5)	3.0 (2.3-4.0)	0.5 (0.1–1.3)	0.0008	0.1	0.1	0.1	0.6	1.3	1.7	0.4

Data are expressed as median (IQR). *P*-value is comparison of C19 with the combined NHK group using Mann-Whitney test. ANGPT2, angiopoietin-2; THBD, thrombomodulin

**Table:** 

Hospital time (days)	Reference range	All patients	C19-1	C19-2	C19-3	C19-4	C19-5	C19-6	C19-7
		11 (6–22)	6	20	20	7	12	4	26
History of HT, DM, CKD, tumor, RA, TE, COPD			HT COPD	RA	N	N	HT Tumor TE	HT DM CKD5	HT DM TE COPD
CRP (mg/l)	< 5 mg/l	141 (59–339)	97	188	29	176	473	76	335
IL6 (ng/l)	< 7.0 ng	628 (82-4795)	78	138	8639	258	503	*unk*	5104
D-dimer (mg/l FEU)	< 0.5	4.7 (1.4–16.0)	3.7	2.4	> 40	24.0	1.7	1.6	2.2
Fibrinogen	2.0-4.2	6.5 (4.5–9.5)	4.3	> 9.0	> 9	4.2	< 9.0	4.1	> 9
Creatinine (µmol/l)	F:45–90, M: 60–105	185 (102–335)	170	138	159	69	320	617	134
eGFR (mL/min/1,73 m^2^)	Age > 65: 50–90	27 (15–178)	30	23	33	93	20	10	25
Thrombi/mm^2^	0.8 (0-2.2)	14.7 (6.9–22.5)	15.2	27.8	17.9	1.9	7.6	12.9	19.6
Tubular health (% nuclear area)	9.8 (8.4–11.5)	0.9 (0.3–2.5)	0.9	0.9	0.2	1.3	1.5	5.6	0.2
Glomerulosclerosis (%)	1.3 (0-3.4)	14.0 (3.4–24.6)	6.5	19.8	0.9	9.9	34.9	18.7	7.3
Glomerular size (x10^3^ µm^2^)	20 (15–25)	26 (21–32)	23	18	35	31	24	25	28
Tubulointerstitial fibrosis (%)	6.9 (0.8–56.9)	34.0 (21.9–52.9)	27.7	27.9	37.6	22.6	66.5	61.6	19.7
THBD glomeruli (%)	2.4 (1.5–3.8)	0.9 (0.4–2.1)	0.6	0.8	0.1	1.4	1.7	2.2	1.2
THBD cortex (%)	1.7 (0.9–3.4)	0.4 (0.1–1.3)	0.1	0.1	0.1	0.6	1.3	1.7	0.4
Antivirals			Y	Y					Y

### Renal thrombosis is common and associated with COVID-19-AKI

**Fig. 2:**
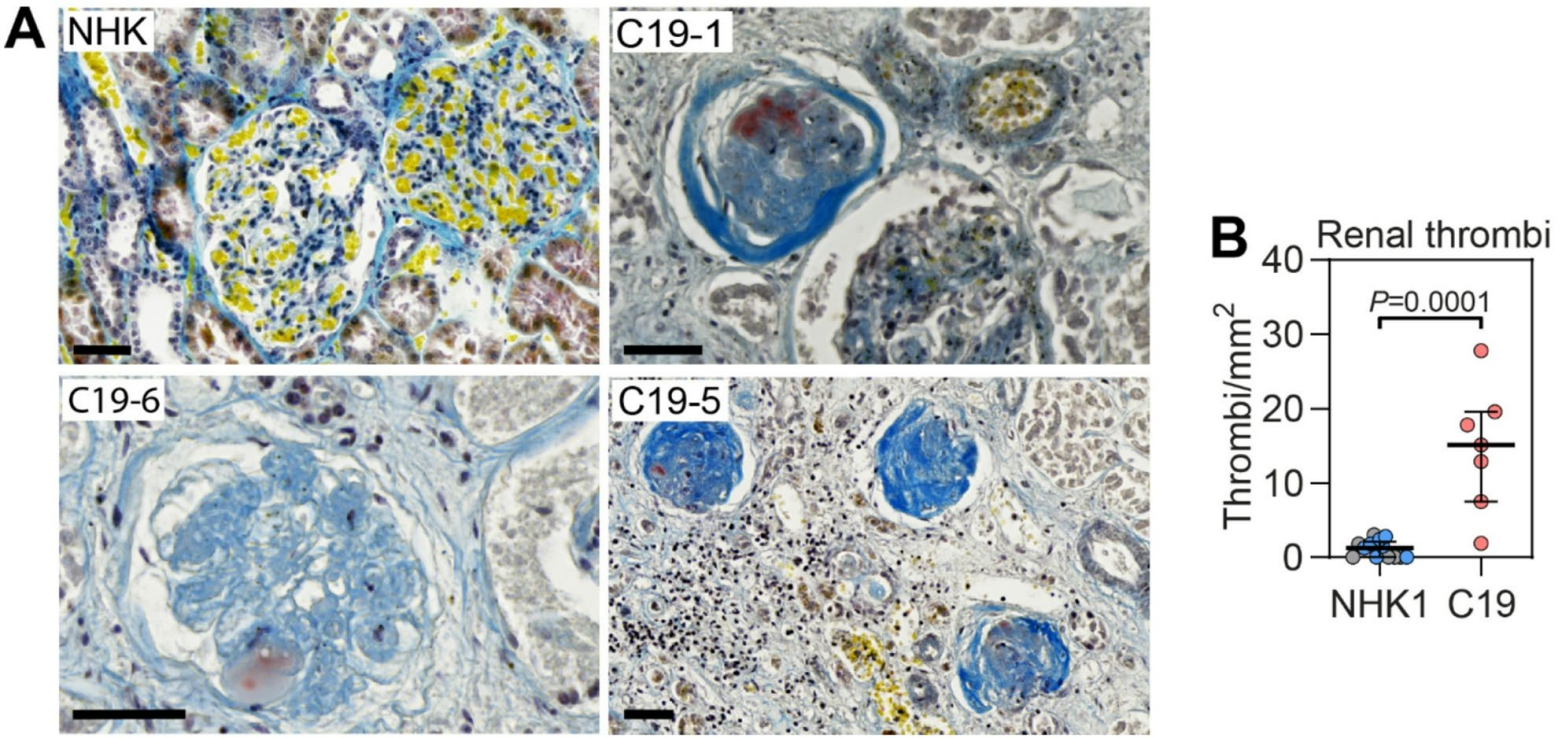
Significant renal thrombosis in critically ill COVID-19 patients. (**A**) Martius Scarlet Blue (MSB) staining of kidney tissue from critically ill COVID-19 patients (C19) and normal human kidney (NHK). MSB stains fibrin (red), fresh fibrin (yellow), erythrocytes (yellow), and connective tissue (blue). Original magnification x200, scale bars = 50 μm. (**B**) Quantification of thrombi from MSB stained tissue expressed as thrombi/mm^2^, in normal human kidney (NHK) from nephrectomies (*n* = 6, grey), postmortem controls (*n* = 7, blue) and COVID-19 patients (C19, *n* = 7, red). Original magnification x400, scale bars = 50 μm. Data are presented as median (IQR). Statistical analysis was performed using the Mann Whitney test

Renal thrombosis was assessed using Martius Scarlet Blue staining of postmortem kidney samples. Thrombi were identified in both glomerular and peritubular capillaries, with a mean density of 15.2 thrombi/mm² in COVID-19 kidney samples, significantly higher than the 1.3 thrombi/mm² observed in control tissue (*P* < 0.0001; Fig. 2; Table 2). One patient (C19-4) presented with only 1.9 thrombi/mm², a value comparable to controls. Notably, this patient was the only one without AKI, with normal P-creatinine (69 µmol/L) and eGFR (93 ml/min/1.73 m²) (Table 1).

### Decreased renal THBD expression suggests impaired local anticoagulation

**Fig. 3:**
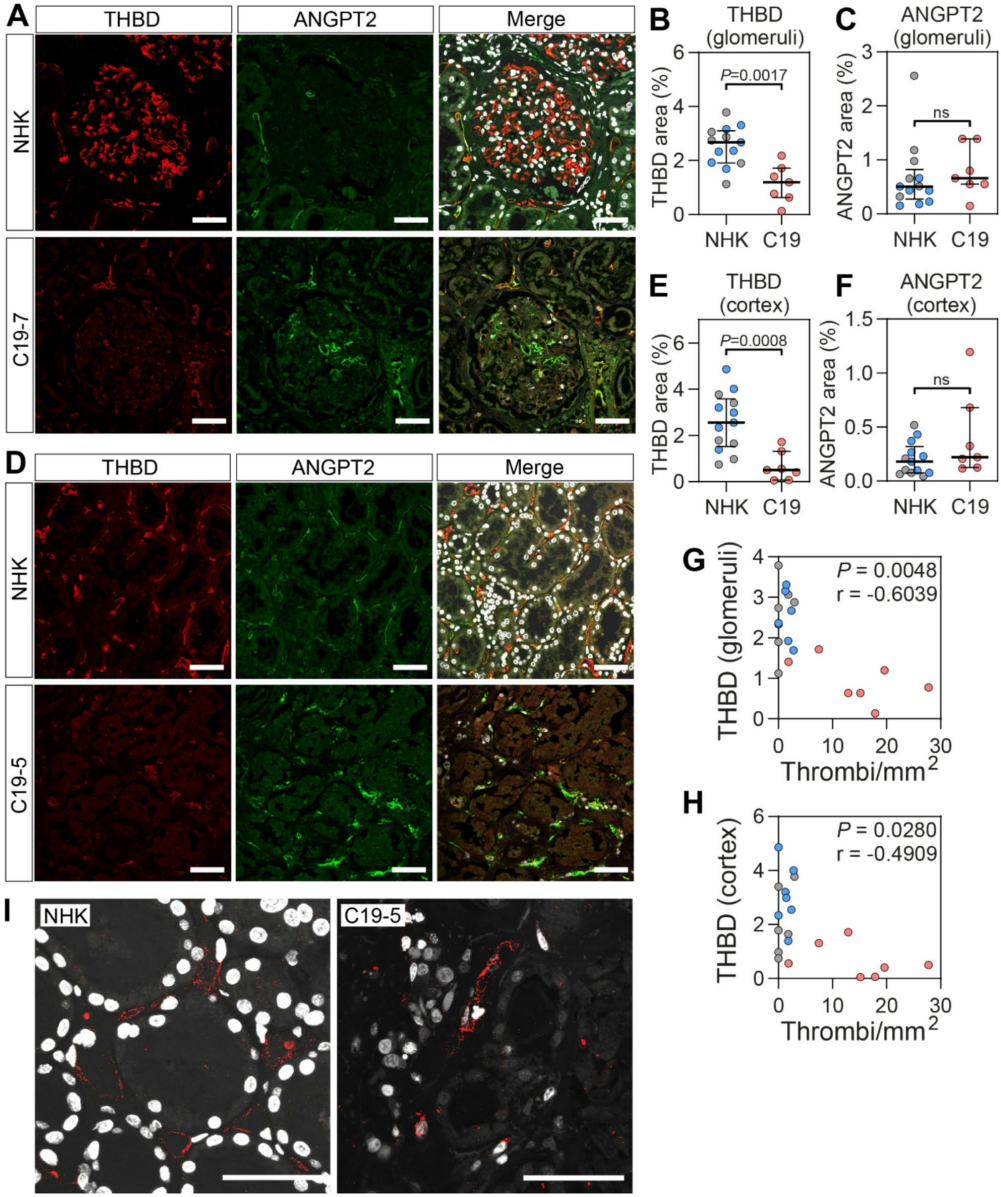
Renal thrombomodulin and angiopoietin-2 expression in critically ill COVID-19 patients. Representative images of immunohistochemistry and quantification of thrombomodulin (THBD) and angiopoietin-2 (ANGPT2) protein expression in (**A-C**) glomeruli and (**D**-**F**) cortex of normal human kidneys (NHK) from nephrectomies (*n* = 6, grey), postmortem controls (*n* = 7, blue) and kidneys from COVID-19 patients (C19, *n* = 7, red). (**G**,** H**) Correlation between THBD protein expression and thrombi. (**I**) Proximity ligation assay showing binding of THBD and ANGPT2 in NHK and COVID-19 kidneys. Original magnification x400, scale bars = 50 μm. Data are presented as median (IQR). Statistical analysis was performed using the Mann Whitney test

Immunostaining of kidney tissue revealed significantly reduced protein expression of THBD in COVID-19 patients, both in peritubular and glomerular capillaries, compared to controls (Fig. 3; Table 2). In peritubular capillaries, THBD expression decreased by 78%, from 2.6% in control kidneys to 0.5% in COVID-19 kidneys (*P* < 0.0008). Similarly, THBD expression in glomerular capillaries decreased from 2.7% to 0.8% (*P* < 0.0017).

The protein expression of angiopoietin-2 (ANGPT2) was also evaluated. In contrast to THBD, ANGPT2 levels showed no significant differences between COVID-19 and control kidneys in either peritubular or glomerular capillaries (Fig. 3; Table 2). As THBD protein expression was reduced in COVID-19, we investigated the correlation between THBD and the number of thrombi in both cortex and glomeruli. In both cases there was a significant correlation between the number of thrombi and THBD protein expression (Fig. 3G, H). To investigate potential interactions between THBD and ANGPT2, a proximity ligation assay (PLA) was employed, capable of detecting protein-protein interactions at a distance of < 40 nm. THBD–ANGPT2 complexes were observed in peritubular capillaries (Fig. 3I); however, due to signal variability, no definitive conclusions could be drawn regarding differential expression between COVID-19 and control tissues.

### Glomerulosclerosis and glomerular size are unchanged in COVID-19-AKI

**Fig. 4:**
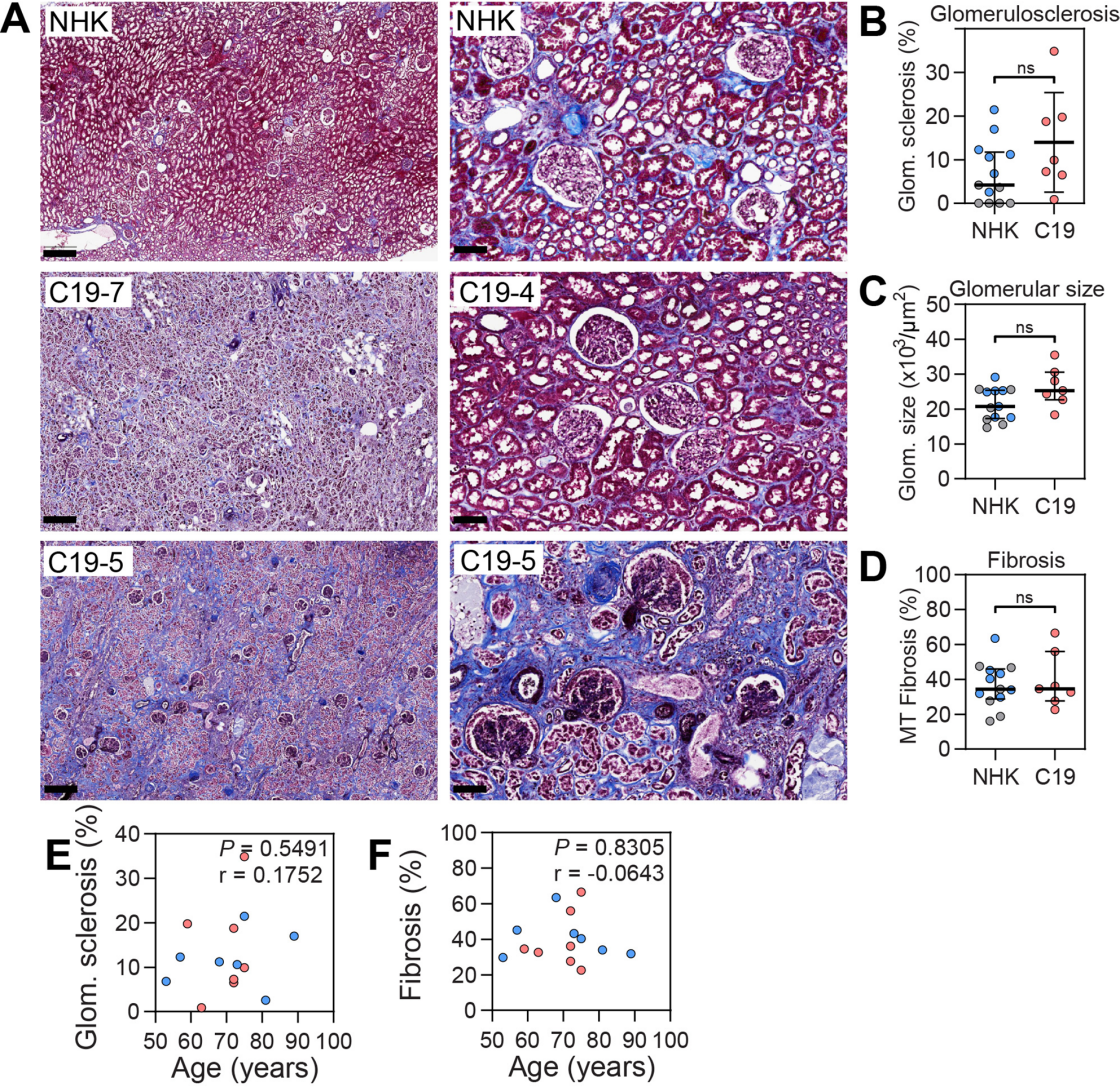
Glomerulosclerosis, glomerular size, and fibrosis in critically ill COVID-19 patients. (**A**) Representative images of Masson trichrome staining of kidneys from normal human kidney (NHK) and COVID-19 patients (C19). Original magnification x200, scale bars = 400 μm (left images) and 100 μm (right images). Quantification of (**B**) glomerulosclerosis and (**C**) glomerular size from MSB-stained sections and (**D**) tubulointerstitial fibrosis from Masson trichrome stained sections from normal human kidney (NHK) from nephrectomies (*n* = 6, grey), postmortem controls (*n* = 7, blue) and kidneys from COVID-19 patients (C19, *n* = 7, red). (**E**,** F**) Correlation between glomerular sclerosis and tubulointerstitial fibrosis with age, respectively. Data are presented as median (IQR). Statistical analysis was performed using the Mann Whitney test

Masson trichrome staining was used to investigate glomerulosclerosis, glomerular size and tubulointerstitial fibrosis in kidneys from COVID-19 patients and controls. All glomeruli (> 4000) in the kidneys were evaluated, and glomerulosclerosis was expressed as a percentage of glomerulosclerotic glomeruli/total glomeruli. No significant differences were found between groups, but it should be noted that several patients in both groups had considerable glomerulosclerosis (Fig. 4A, B; Table 2). Patient C19-5 with a glomerulosclerosis rate of 34.9% had no previous diagnosis of kidney disease. Two other patients, C19-2 and C19-5, had high degrees of glomerulosclerosis as well, at 19.8% and 18.7%, respectively. Patient C19-5 had a history of diabetes and CKD5 which can explain the high degree of glomerulosclerosis. Patient C19-2, on the other hand, had rheumatoid arthritis but no previous history of kidney disease.

During the assessment of glomerulosclerosis, enlarged glomeruli were observed in several patients, prompting a quantitative comparison of glomerular area between groups. The mean glomerular area in control kidneys was 20.7 × 10³ µm², consistent with prior reports [32, 33]. Glomeruli from COVID-19 kidneys had a median area of 25.3 × 10³ µm² (Fig. 4B, C; Table 2) which was not significantly different from controls. The increase in size was most prominent in patients C19-3, C19-4, and C19-7. Of these, patients C19-3 and C19-4 had no documented comorbidities, whereas C19-7 had type 2 diabetes mellitus, a condition known to cause glomerular hypertrophy [33].

Analysis of tubulointerstitial fibrosis revealed substantial fibrotic changes in the cortex of both control and COVID-19 kidneys with no significant difference between groups. Patients C19-5 and C19-6 presented with the highest degrees of tubulointerstitial fibrosis, measured at 66.5% and 61.6%, respectively. The extent of fibrosis in C19-5 is consistent with the patient’s history of CKD stage 5. In contrast, patient C19-6 had no prior history of kidney disease. Glomerulosclerosis and tubulointerstitial fibrosis increase with age [34, 35], however, no correlation with age was found in the current cohort (Fig. 4E, F).

In summary, histopathological examination of kidney tissue from seven critically ill COVID-19 patients revealed extensive tubular necrosis in all cases. AKI was present in six patients, all of whom also demonstrated widespread renal microthrombosis. Other renal pathologies, including glomerulosclerosis and tubulointerstitial fibrosis, showed variable expression among individuals and were not significantly different between controls and COVID-19 patients. Notably, decreased THBD protein expression suggests a loss of anticoagulative protection may contribute to a higher risk of microthrombosis.

## Discussion

We hypothesized that microthrombi could contribute to AKI in critically ill COVID-19 patients. This study supports our hypothesis and demonstrates marked renal microthrombosis and endothelial dysfunction characterized by significantly decreased expression of anticoagulant thrombomodulin.

Consistent with prior reports, decreased kidney function was observed in a majority of patients [1–4]. Notably, nuclear area quantification revealed a profound reduction in tubular epithelial nuclear area, indicative of extensive tubular cell loss, consistent with other studies [7, 8, 36]. This tubular necrosis likely contributes to the functional decline observed in these patients, corroborated by elevated plasma creatinine and reduced eGFR. The detection of SARS-CoV-2 within tubular segments and ACE2 expression on tubular epithelium supports a mechanism of direct viral cytopathic effect, aligning with previous findings implicating SARS-CoV-2 in direct renal epithelial infection [37, 38].

A hallmark of our findings is the widespread presence of thrombi in glomerular and peritubular capillaries in COVID-19 kidneys, with a thrombus density 10-fold higher than in controls. Other studies have reported similar frequences of renal microthrombi [7, 9]. Three other studies have reported incidences in the range of 4.5–14% [8, 10, 36]. The patient records of clinical data showed that all COVID-19 patients in the current study had increased D-dimer levels, and either borderline high or increased fibrinogen, in line with a hypercoagulative state. The patient without AKI exhibited a thrombotic burden similar to healthy controls, providing a rational to investigate the association of thrombotic burden and AKI in a larger cohort. These data reinforce prior autopsy findings and emerging clinical studies that identify COVID-19 as a prothrombotic state with high microvascular involvement [6–10].

COVID-19 induces endothelial activation and injury, and microthrombosis reflect disturbances in endothelial integrity, platelet activation, coagulation, and immune-driven pathways. This shift toward a pro-coagulant phenotype includes increased tissue factor expression, excess von Willebrand factor release (2–4). Immunothrombotic mechanisms, including neutrophil extracellular traps and complement activation, add potent pro-thrombotic stimuli highly characteristic of COVID-19 [11, 12]. Parallel platelet hyperreactivity, exacerbated by inflammatory cytokines, high-shear exposure to abundant von Willebrand factor, and immune interactions, amplifies microvascular thrombus formation [39, 40].

Reduced expression of THBD in glomerular and peritubular capillaries points toward a compromised local anticoagulant environment. Loss of THBD reduces protein C activation and removes a key anticoagulant restraint on thrombin generation [15, 16] and may predispose to microthrombi formation. The reduction in THBD expression aligns with our previous work demonstrating that angiopoietin-2 (ANGPT2) promotes thrombomodulin shedding and inhibits its anticoagulant function [13]. Loss of THBD from lung endothelium has been shown in COVID-19 [19, 41], but to our knowledge no data are available for the kidney in COVID-19.

ANGPT2 levels in renal tissue were not significantly elevated compared to controls. In agreement with this, Volbeda et al. described a similar *ANGPT2* expression pattern in renal postmortem biopsies from COVID-19 patients with AKI compared to controls [7]. As circulating levels of ANGPT2 are known to be markedly increased in critically ill COVID-19 patients [13, 17–21], the renal endothelium could still be exposed to high levels. Proximity ligation assays identified ANGPT2–THBD complexes in peritubular capillaries, suggesting a possible functional interaction at the endothelial surface. However, signal variability precluded definitive conclusions about altered ANGPT2–THBD complex formation in COVID-19 tissue. As we did not measure circulating levels of ANGPT2 in the current study, the role of plasma ANGPT2 remains to be clarified in future studies.

Glomerulosclerosis and tubulointerstitial fibrosis were considerable in both groups and highly variable in both controls and COVID-19 patients. While some COVID-19 patients had known risk factors for glomerulosclerosis including diabetes and CKD, others lacked prior renal diagnoses. The presence of substantial glomerulosclerosis and tubulointerstitial fibrosis also in controls, raised the question of an association with age as previously described [35]. We found no association with age in the current cohort, perhaps due to the small sample size.

This study is limited by the small sample size and the retrospective nature of clinical data collection. Control tissues were obtained from nephrectomy specimens and postmortem tissue from non-COVID-19 patients, and full clinical histories for those individuals were unavailable, which may confound comparisons. Furthermore, while postmortem tissues provide valuable insights into disease pathogenesis, they represent a single timepoint at end-stage disease and cannot capture the temporal dynamics of injury and repair. Time between death and autopsy was 4–7 days and it cannot be ruled out that autolysis occurred, especially in tubular epithelial cells, however, the presence of better-preserved tubular cells in postmortem control tissue suggest that at least in part COVID-19 contributes to acute tubular necrosis.

**Fig. 5:**
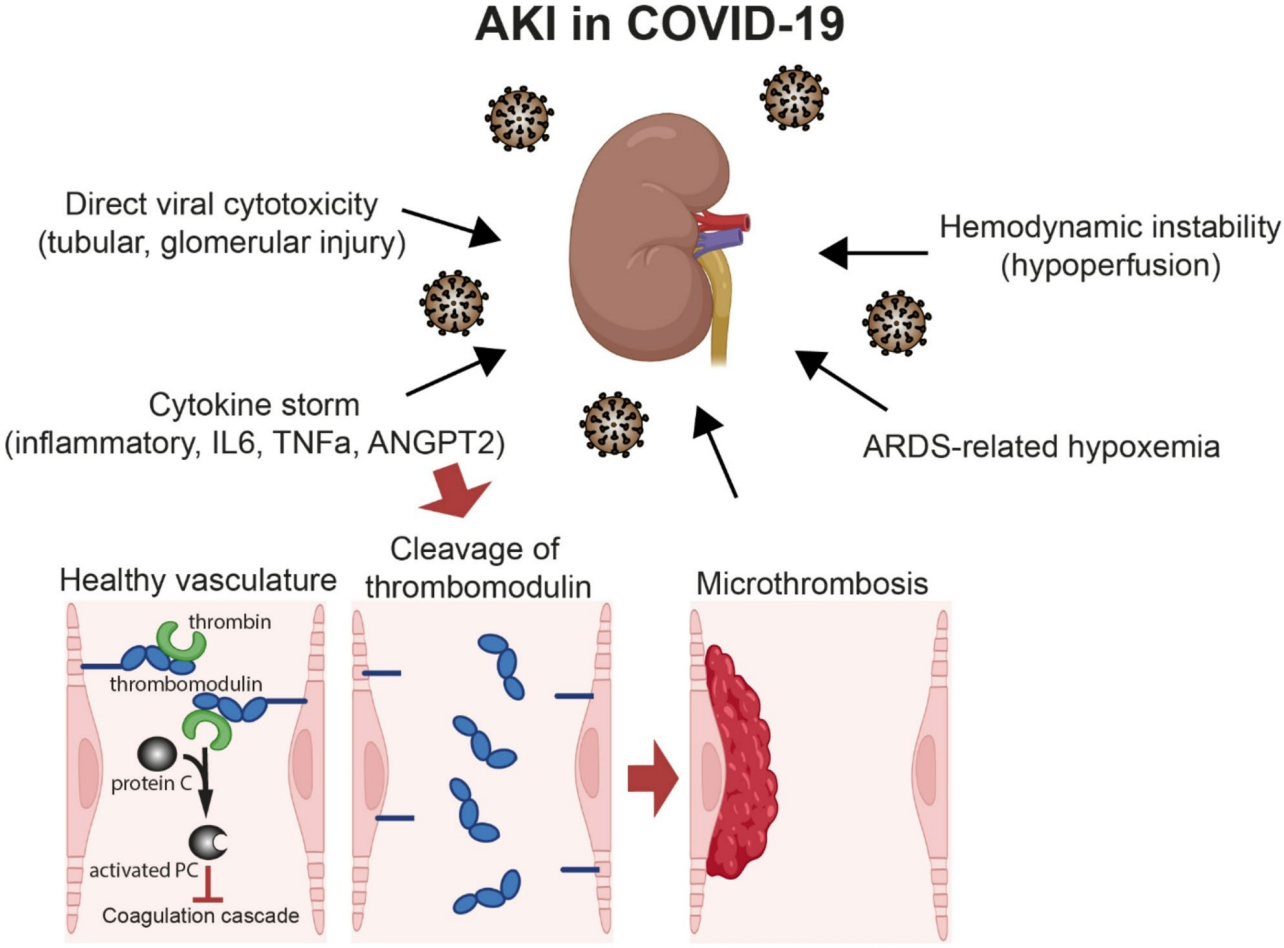
A schematic illustration of several pathways leading to AKI. As the summary of the current study, healthy blood vessels have anticoagulant activity mediated by THBD. In COVID-19, elevated circulating inflammatory cytokines including ANGPT2, promote cleavage of THBD from the endothelial surface. Loss of endothelial THBD diminishes the local anticoagulant capacity resulting in a markedly increased risk of microthrombosis and subsequent loss of kidney function

In summary, this study demonstrates that acute kidney injury in critically ill COVID-19 patients is closely associated with extensive renal microthrombosis, profound tubular epithelial injury, and marked endothelial dysfunction (Fig. 5). Tubular necrosis, supported by reduced nuclear area and detection of SARS-CoV-2 within tubular segments, indicates a contribution from direct viral cytopathic effects. A striking increase in glomerular and peritubular capillary thrombi, accompanied by elevated D-dimer and fibrinogen levels, highlights a strong link between intrarenal thrombosis and impaired kidney function. Notably, we observed significantly reduced expression of THBD in renal microvessels, pointing to a compromised local anticoagulant environment that may predispose to microthrombus formation. Together, these findings support a model in which the combined effects of viral tubular injury and loss of endothelial anticoagulant signaling contribute to widespread microvascular thrombosis and COVID-19–associated AKI.

## Conclusions

In conclusion, our findings show that that likely direct viral tubular injury, together with widespread renal microthrombosis and endothelial dysfunction with reduced thrombomodulin expression, contributes to the development of COVID-19–associated AKI.

## Data Availability

The datasets used and/or analysed during the current study are available from the corresponding author on reasonable request.

## Abbreviations

SARS-CoV-2: Severe acute respiratory syndrome coronavirus 2
AKI: Acute kidney injury
CKD: Chronic kidney disease
2-ANGPT2: Angiopoietin
THBD: Thrombomodulin
1-ANGPT1: Angiopoietin

## References

[1] Schaubroeck H, Vandenberghe W, Boer W, Boonen E, Dewulf B, Bourgeois C, Dubois J, Dumoulin A, Fivez T, Gunst J, et al. Acute kidney injury in critical COVID-19: a multicenter cohort analysis in seven large hospitals in Belgium. Crit Care (London England). 2022;26(1):225. 10.1186/s13054-022-04086-x PMC9310674 35879765

[2] Lumlertgul N, Pirondini L, Cooney E, Kok W, Gregson J, Camporota L, Lane K, Leach R, Ostermann M. Acute kidney injury prevalence, progression and long-term outcomes in critically ill patients with COVID-19: a cohort study. Ann Intensive Care. 2021;11(1):123. 34357478 10.1186/s13613-021-00914-5 PMC8343342

[3] Silver SA, Beaubien-Souligny W, Shah PS, Harel S, Blum D, Kishibe T, Meraz-Munoz A, Wald R, Harel Z. The prevalence of acute kidney injury in patients hospitalized with COVID-19 infection: A systematic review and Meta-analysis. Kidney Med. 2021;3(1):83–e9881. 33319190 10.1016/j.xkme.2020.11.008 PMC7723763

[4] Sanchez AV, Perez AN, Perez-Carrasco M, Sonet MT, Buendia YD, Ballujera PO, Lopez MR, Riera JS, Olmo-Isasmendi A, Torra EV, et al. Acute kidney injury in critically ill patients with COVID-19: the AKICOV multicenter study in Catalonia. PLoS ONE. 2023;18(4):e0284248. 37058544 10.1371/journal.pone.0284248 PMC10104297

[5] Khwaja A. KDIGO clinical practice guidelines for acute kidney injury. Nephron Clin Pract. 2012;120(4):c179–184. 22890468 10.1159/000339789

[6] Nadim MK, Forni LG, Mehta RL, Connor MJ Jr., Liu KD, Ostermann M, Rimmele T, Zarbock A, Bell S, Bihorac A, et al. COVID-19-associated acute kidney injury: consensus report of the 25th acute disease quality initiative (ADQI) workgroup. Nat Rev Nephrol. 2020;16(12):747–64. 33060844 10.1038/s41581-020-00356-5 PMC7561246

[7] Volbeda M, Jou-Valencia D, van den Heuvel MC, Knoester M, Zwiers PJ, Pillay J, Berger SP, van der Voort PHJ, Zijlstra JG, van Meurs M, et al. Comparison of renal histopathology and gene expression profiles between severe COVID-19 and bacterial sepsis in critically ill patients. Crit Care (London England). 2021;25(1):202. 10.1186/s13054-021-03631-4 PMC8190989 34112226

[8] Menter T, Haslbauer JD, Nienhold R, Savic S, Hopfer H, Deigendesch N, Frank S, Turek D, Willi N, Pargger H, et al. Postmortem examination of COVID-19 patients reveals diffuse alveolar damage with severe capillary congestion and variegated findings in lungs and other organs suggesting vascular dysfunction. Histopathology. 2020;77(2):198–209. 32364264 10.1111/his.14134 PMC7496150

[9] Duarte-Neto AN, Monteiro RAA, da Silva LFF, Malheiros D, de Oliveira EP, Theodoro-Filho J, Pinho JRR, Gomes-Gouvea MS, Salles APM, de Oliveira IRS, et al. Pulmonary and systemic involvement in COVID-19 patients assessed with ultrasound-guided minimally invasive autopsy. Histopathology. 2020;77(2):186–97. 32443177 10.1111/his.14160 PMC7280721

[10] Nicolai L, Leunig A, Brambs S, Kaiser R, Weinberger T, Weigand M, Muenchhoff M, Hellmuth JC, Ledderose S, Schulz H, et al. Immunothrombotic dysregulation in COVID-19 pneumonia is associated with respiratory failure and coagulopathy. Circulation. 2020;142(12):1176–89. 32755393 10.1161/CIRCULATIONAHA.120.048488 PMC7497892

[11] Golmai P, Larsen CP, DeVita MV, Wahl SJ, Weins A, Rennke HG, Bijol V, Rosenstock JL. Histopathologic and ultrastructural findings in postmortem kidney biopsy material in 12 patients with AKI and COVID-19. J Am Soc Nephrol. 2020;31(9):1944–7. 32675304 10.1681/ASN.2020050683 PMC7461690

[12] Yende S, Parikh CR. Long COVID and kidney disease. Nat Rev Nephrol. 2021;17(12):792–3. 34504319 10.1038/s41581-021-00487-3 PMC8427150

[13] Hultström M, Fromell K, Larsson A, Quaggin S, Betsholtz C, Frithiof R, Lipcsey M, Jeansson M. Elevated Angiopoietin-2 inhibits thrombomodulin-mediated anticoagulation in critically ill COVID-19 patients. MedRxiv. 2021. 10.1101/2021.1101.1113.21249429. 10.3390/biomedicines10061333 PMC9220312 35740360

[14] Daly C, Qian X, Castanaro C, Pasnikowski E, Jiang X, Thomson BR, Quaggin SE, Papadopoulos N, Wei Y, Rudge JS, et al. Angiopoietins bind thrombomodulin and inhibit its function as a thrombin cofactor. Sci Rep. 2018;8(1):505. 29323190 10.1038/s41598-017-18912-8 PMC5765006

[15] Esmon NL, Owen WG, Esmon CT. Isolation of a membrane-bound cofactor for thrombin-catalyzed activation of protein C. J Biol Chem. 1982;257(2):859–64. 6895633

[16] Esmon CT. Inflammation and the activated protein C anticoagulant pathway. Semin Thromb Hemost. 2006;32(Suppl 1):49–60. 16673266 10.1055/s-2006-939554

[17] Villa E, Critelli R, Lasagni S, Melegari A, Curatolo A, Celsa C, Romagnoli D, Melegari G, Pivetti A, Di Marco L, et al. Dynamic angiopoietin-2 assessment predicts survival and chronic course in hospitalized patients with COVID-19. Blood Adv. 2021;5(3):662–73. 33560382 10.1182/bloodadvances.2020003736 PMC7876870

[18] Smadja DM, Guerin CL, Chocron R, Yatim N, Boussier J, Gendron N, Khider L, Hadjadj J, Goudot G, Debuc B, et al. Angiopoietin-2 as a marker of endothelial activation is a good predictor factor for intensive care unit admission of COVID-19 patients. Angiogenesis 2020. 10.1007/s10456-020-09730-0 PMC7250589 32458111

[19] Schmaier AA, Pajares Hurtado GM, Manickas-Hill ZJ, Sack KD, Chen SM, Bhambhani V, Quadir J, Nath AK, Collier AY, Ngo D, et al. Tie2 activation protects against prothrombotic endothelial dysfunction in COVID-19. JCI Insight 2021. 10.1172/jci.insight.151527 PMC8564889 34506304

[20] Rovas A, Osiaevi I, Buscher K, Sackarnd J, Tepasse P-R, Fobker M, Kühn J, Braune S, Göbel U, Thölking G, et al. Microvascular dysfunction in COVID-19: the MYSTIC study. Angiogenesis. 2020. 10.1007/s10456-020-09753-7 PMC7556767 33058027

[21] Francischetti IMB, Toomer K, Zhang Y, Jani J, Siddiqui Z, Brotman DJ, Hooper JE, Kickler TS. Upregulation of pulmonary tissue factor, loss of thrombomodulin and immunothrombosis in SARS-CoV-2 infection. EClinicalMedicine. 2021;39:101069. 34377969 10.1016/j.eclinm.2021.101069 PMC8342934

[22] Pietila R, Marks-Hultstrom A, He L, Nanavazadeh S, Quaggin SE, Betsholtz C, Jeansson M. TIE2 activation by antibody-clustered endogenous angiopoietin-2 prevents capillary loss and fibrosis in experimental kidney disease. J Clin Invest. 2025;135(21). 10.1172/JCI190286 PMC12578391 40952790

[23] Koh GY. Orchestral actions of angiopoietin-1 in vascular regeneration. Trends Mol Med. 2013;19(1):31–9. 23182855 10.1016/j.molmed.2012.10.010

[24] Mandriota SJ, Pepper MS. Regulation of angiopoietin-2 mRNA levels in bovine microvascular endothelial cells by cytokines and hypoxia. Circ Res. 1998;83(8):852–9. 9776732 10.1161/01.res.83.8.852

[25] Fiedler U, Scharpfenecker M, Koidl S, Hegen A, Grunow V, Schmidt JM, Kriz W, Thurston G, Augustin HG. The Tie-2 ligand angiopoietin-2 is stored in and rapidly released upon stimulation from endothelial cell Weibel-Palade bodies. Blood. 2004;103(11):4150–6. 14976056 10.1182/blood-2003-10-3685

[26] Jang C, Koh YJ, Lim NK, Kang HJ, Kim DH, Park SK, Lee GM, Jeon CJ, Koh GY. Angiopoietin-2 exocytosis is stimulated by sphingosine-1-phosphate in human blood and lymphatic endothelial cells. Arterioscler Thromb Vasc Biol. 2009;29(3):401–7. 19112163 10.1161/ATVBAHA.108.172676

[27] Daly C, Wong V, Burova E, Wei Y, Zabski S, Griffiths J, Lai KM, Lin HC, Ioffe E, Yancopoulos GD, et al. Angiopoietin-1 modulates endothelial cell function and gene expression via the transcription factor FKHR (FOXO1). Genes Dev. 2004;18(9):1060–71. 15132996 10.1101/gad.1189704 PMC406295

[28] Ghosh CC, David S, Zhang R, Berghelli A, Milam K, Higgins SJ, Hunter J, Mukherjee A, Wei Y, Tran M, et al. Gene control of tyrosine kinase TIE2 and vascular manifestations of infections. Proc Natl Acad Sci U S A. 2016;113(9):2472–7. 26884170 10.1073/pnas.1519467113 PMC4780619

[29] Lukasz A, Hillgruber C, Oberleithner H, Kusche-Vihrog K, Pavenstadt H, Rovas A, Hesse B, Goerge T, Kumpers P. Endothelial glycocalyx breakdown is mediated by angiopoietin-2. Cardiovascular Res. 2017;113(6):671–80. 10.1093/cvr/cvx023 28453727

[30] Drost CC, Rovas A, Kusche-Vihrog K, Van Slyke P, Kim H, Hoang VC, Maynes JT, Wennmann DO, Pavenstadt H, Linke W, et al. Tie2 activation promotes protection and reconstitution of the endothelial glycocalyx in human sepsis. Thromb Haemost. 2019;119(11):1827–38. 31493777 10.1055/s-0039-1695768

[31] Diao B, Wang C, Wang R, Feng Z, Zhang J, Yang H, Tan Y, Wang H, Wang C, Liu L, et al. Human kidney is a target for novel severe acute respiratory syndrome coronavirus 2 infection. Nat Commun. 2021;12(1):2506. 33947851 10.1038/s41467-021-22781-1 PMC8096808

[32] Grande JP, Helgeson ES, Matas AJ. Correlation of glomerular size with Donor-Recipient factors and with response to injury. Transplantation. 2021;105(11):2451–60. 33273317 10.1097/TP.0000000000003570 PMC8166916

[33] Abdi R, Slakey D, Kittur D, Racusen LC. Heterogeneity of glomerular size in normal donor kidneys: impact of race. Am J Kidney Dis. 1998;32(1):43–6. 9669422 10.1053/ajkd.1998.v32.pm9669422

[34] Hodgin JB, Bitzer M, Wickman L, Afshinnia F, Wang SQ, O’Connor C, Yang Y, Meadowbrooke C, Chowdhury M, Kikuchi M, et al. Glomerular aging and focal global glomerulosclerosis: A podometric perspective. J Am Soc Nephrol. 2015;26(12):3162–78. 26038526 10.1681/ASN.2014080752 PMC4657829

[35] Yang HC, Fogo AB. Fibrosis and renal aging. Kidney Int Suppl (2011). 2014;4(1):75–8. 26312154 10.1038/kisup.2014.14 PMC4536965

[36] Santoriello D, Khairallah P, Bomback AS, Xu K, Kudose S, Batal I, Barasch J, Radhakrishnan J, D’Agati V, Markowitz G. Postmortem kidney pathology findings in patients with COVID-19. J Am Soc Nephrol. 2020;31(9):2158–67. 32727719 10.1681/ASN.2020050744 PMC7461662

[37] Reich HN, Oudit GY, Penninger JM, Scholey JW, Herzenberg AM. Decreased glomerular and tubular expression of ACE2 in patients with type 2 diabetes and kidney disease. Kidney Int. 2008;74(12):1610–6. 19034303 10.1038/ki.2008.497

[38] Fan C, Lu W, Li K, Ding Y, Wang J. ACE2 expression in kidney and testis may cause kidney and testis infection in COVID-19 patients. Front Med. 2021;7. 10.3389/fmed.2020.563893 PMC7838217 33521006

[39] Nieswandt B, Pleines I, Bender M. Platelet adhesion and activation mechanisms in arterial thrombosis and ischaemic stroke. J Thromb Haemost. 2011;9(Suppl 1):92–104. 21781245 10.1111/j.1538-7836.2011.04361.x

[40] Comer SP, Cullivan S, Szklanna PB, Weiss L, Cullen S, Kelliher S, Smolenski A, Murphy C, Altaie H, Curran J, et al. COVID-19 induces a hyperactive phenotype in Circulating platelets. PLoS Biol. 2021;19(2):e3001109. 33596198 10.1371/journal.pbio.3001109 PMC7920383

[41] Won T, Wood MK, Hughes DM, Talor MV, Ma Z, Schneider J, Skinner JT, Asady B, Goerlich E, Halushka MK, et al. Endothelial thrombomodulin downregulation caused by hypoxia contributes to severe infiltration and coagulopathy in COVID-19 patient lungs. EBioMedicine. 2022;75:103812. 35033854 10.1016/j.ebiom.2022.103812 PMC8756077

